# Oocyte development: it’s all about quality

**DOI:** 10.1016/j.rbmo.2025.104804

**Published:** 2025-04-01

**Authors:** Richard A Anderson, Adele L Marston, Evelyn E Telfer

**Affiliations:** 1 Centre for Reproductive Health, Institute for Repair and Regeneration, University of Edinburgh, Edinburgh, United Kingdom; 2 The Wellcome Centre for Cell Biology, Institute of Cell Biology, University of Edinburgh, Edinburgh, United Kingdom; 3 Institute of Cell Biology, University of Edinburgh, Edinburgh, United Kingdom

**Keywords:** oocyte, ovarian development, meiosis, aneuploidy, cohesin

## Abstract

Mammalian fertility is dependent on the production of an oocyte capable of fertilisation and supporting early embryo development. This requires both cytoplasmic and nuclear, i.e. chromosomal, competence, processes that were initiated decades prior to ovulation. Current demographic changes with delayed motherhood are increasingly in conflict with these biological processes. In this brief review we highlight the key stages in oocyte development, and recent findings that continue to inform on how the oocyte is able to maintain function over such a prolonged period. These include minimising oocyte damage through the production of reactive oxygen species, the importance of intercellular communication with surrounding somatic cells, and the molecular mechanisms that underpin the fidelity of chromosome cohesion and then separation at the resumption of meiosis. Some of these are already approaching clinical testing and interventions, with in the coming years new approaches potentially being able to ‘put back the clock’ to improve oocyte quality.

The ovary performs a series of remarkable feats by remaining relatively quiescent for a decade, then subsequently delivering approximately two and a half decades of potential fertility within four decades of endocrine function (figure 1). Both key aspects of its function will therefore have essentially ceased by the age of 50, and with increasing female longevity in tandem with demographic changes resulting in many women starting their families at a later age than historically was the case, this means that ovaries are increasingly not delivering what women need of them in the modern world (Anderson and Hickey, 2023). This early cessation of key functions contrasts with the lifelong function of the body’s other organ systems, including the uterus, and also contrasts with the lifelong endocrine and fertile function of the testes. Dealing with the consequences of the decline in ovarian function in both number and quality of gametes is the bread and butter of the modern fertility clinic and with therefore much research effort put into optimising and extending both those aspects of its function. Ultimately, however, the achievements of modern medicine have been very limited in this regard other than the development of synthetic steroids half a century ago and subsequently the refinements of gonadotropin purification and synthesis. While the clinical process of IVF, whose origins are being celebrated in this series of short reviews, has been transformational in may regards, its approach to declining oocyte quantity and quality remains to try to overcome them by force of numbers, or through being able to use eggs from a different, younger woman. There are however many exciting areas of research activity that do address these fundamental issues, in increased understanding of the physiological factors that govern regulation of the maintenance of oocyte health within the dormant follicle and during follicle development, the regulation of follicle growth activation itself and the control of ongoing follicle development in competition with other growing follicles, and the molecular mechanisms that underpin the fidelity of delivering a euploid gamete to the awaiting spermatozoa. Many of these are open to therapeutic intervention, the development of which is in some cases underway. The opportunity to bypass all this with artificial gametes is rapidly approaching, but outwith our remit here. In this short review we will consider some of the key aspects of ovarian function that underpin that key aspect of fertility, the development of a competent oocyte.

While a major focus of both research and clinical practice in assisted reproduction hinges on the euploidy or otherwise of the oocyte, one aspect that is physiologically remarkable is that it has its origins long before fertility is required, in early fetal life. Following a period of germ cell proliferation in the later first trimester, driven by incompletely understood signals but involving retinoic acid signalling and RNA binding proteins such as DAZL (Rosario, et al., 2017), the oogonia start to enter meiosis. Unlike in the rodent ovary where this wave of meiotic entry progresses longitudinally along the ovary, in the human this originates in the more central oogonia, with those around the periphery of the ovary remaining mitotic and thus by approximately 15 weeks of development the first primordial follicles are now evident but still surrounded by continuing oogonial proliferation. During this first prophase of meiosis the chromosome cross-overs essential for genetic diversity are established and will require to be held there stably until meiosis resumes at the time of ovulation. This is achieved because the sister chromatids are held together by sister chromatid cohesion that is established along the length of chromosomes as they replicate. Cohesion is mediated by a ring-shaped protein complex called cohesin, which is increasingly understood at the molecular and biochemical level, discussed further below. Genetic mutations in the genes underpinning key cohesin components have been identified in both women and men with defects is gametogenesis, resulting in premature ovarian insufficiency and azoospermia respectively (Beverley, et al., 2021). In addition to these intrinsic factors helping to establish a large pool of potentially competent germ cells, the ovary will also be subject to the environmental influences mediated through the pregnant woman such as environmental chemicals from air, water and food pollution and obesity with these influences therefore potentially impacting the health of the pregnant woman’s grandchildren.

Following all this early critical activity the population of primordial follicles is therefore established before birth. While the existence of oogonia within the postnatal ovary that might have the potential to form primordial follicles subsequently remains the subject of debate in the human, this is clearly established in many species including some mammals. The potential value of being able to stimulate reactivation of these cells in later reproductive life is obvious but it remains an area in reproductive medicine whose debate seems to result in more heat than light.

Studies on the ovary during childhood in the 1970s and indeed earlier had identified significant follicular activity and more recent studies have identified ongoing changes in the ovary during that period that are likely to impact on adult oocyte quality. Morphologically the ovary in childhood contains many primordial follicles with abnormal features, and while these are very common in younger children, constituting perhaps a third of all follicles, they are progressively lost such that those features are never seen in the ovaries of adult women (Anderson, et al., 2014). Functionally, it has long been known that pregnancies in teenage women have a higher prevalence of aneuploidy than those in women in their twenties and light has recently been shed on the molecular basis of this within increased frequency of metaphase I of nondisjunction occurring in oocytes in women under the age of 20, contrasting with the increased aneuploidies in older women which result from premature separation of sister chromatids and reverse segregation (Gruhn, et al., 2019). This may reflect the progressive changes in chromosome cohesion over time, which progressively declines, and thus the nondisjunction seen in younger women may reflect essentially too much cohesion at that point rather than the too little cohesion that underpins the errors of later life, with a ‘sweet spot’ in the 20’s. Further discussion of these changes in later reproductive life can be found below.

Progressive changes in the composition of the ovarian stroma also occur, starting in adolescence and progressing through reproductive life into the post-reproductive period. These changes, particularly in the proportion of collagen and elastin composition of the stroma, result in an increased ovarian stiffness during childhood and adolescence, with again a further increase during the later reproductive years (Ouni, et al., 2020). This is now established to impact the rate of follicular growth activation, with reduced activation of primordial follicles in a stiffer ovarian cortex. An important finding has been the dramatic increase in ovarian fibrosis associated with age, initially identified in mice but now also confirmed to occur in women (Briley, et al., 2016). This is also associated with an increased inflammatory reaction within the ovary, a growing area of research with as yet uncertain implications. It has, however, been demonstrated in a mouse model that drugs that reduce fibrosis and the associated inflammation, whether related to age or indeed to obesity, can increase the fertility of treated older animals (Umehara, et al., 2022). Remarkably, metformin is active in this regard, as are drugs that improve mitochondrial function. This is a very open area for research with potentially immediate clinical benefits.

Primordial follicles are the fundamental developmental structures of the mammalian ovary consisting of an oocyte arrested at Prophase I of meiosis and somatic cells (granulosa) arrested at the G0 stage of the cell cycle (Telfer, et al., 2023), with mutual interdependency throughout follicle growth and oocyte maturation to the point of ovulation. Primordial follicles can (and most are required to) remain dormant for many years before being activated to grow and develop a mature oocyte. Prolonged dormancy presents a significant challenge in maintaining quality as oocytes need to be protected from stressors such as reactive oxygen species (ROS) which are by products of cell metabolism that are highly reactive and cause oxidative damage to cell components. Recent work has demonstrated that dormant oocytes have evolved a mechanism to limit ROS damage by suppressing mitochondrial complex 1, a key generator of ROS within the cell (Rodriguez-Nuevo, et al., 2022). The lack of complex 1 results in less efficient cellular metabolism, but since that is low in primordial follicles, it appears that it is a price well worth paying for the benefit of reduced ROS production. Once follicles are activated to grow ROS becomes a significant factor in affecting oocyte quality and alleviating this has been a key therapeutic target to mitigate the effects of ageing. Additional mechanisms to protect primordial follicles have been uncovered with the discovery that long lived proteins exist within the oocytes and somatic cells of mouse primordial follicles and these act to alleviate damage within the germline (Harasimov, et al., 2024). Some of the proteins identified as being long lived include TWINKLE, a mitochondrial DNA helicase essential for mitochondrial DNA replication, BOP1, essential for ribosome assembly and cohesin-related proteins (Harasimov, et al., 2024).

The pool of primordial follicles is progressively reduced throughout life with activation of growth being a continuous process. The transition from primordial to primary follicles is characterised by the differentiation and proliferation of granulosa cells to form a single layer of cuboidal-shaped cells that surround the oocyte. During follicle development the oocyte undergoes growth and acquires nuclear and cytoplasmic competence which is dependent upon intercellular communication between the growing oocyte and the developing somatic (granulosa) cells. Dynamic bidirectional communications are established between the oocyte and somatic cell compartments, through the establishment of physical connections and the secretion of autocrine and paracrine growth factors with maintenance of these connections being essential for progression through each developmental stage (figure 2). At the onset of follicular growth, intimate intercellular connections are established and narrow cytoplasmic filopodia-like extensions, known as transzonal projections (TZPs), extend from the granulosa cells and traverse the zona pellucida to connect the oocyte plasma membrane. Gap junctions are located at the tips of TZPs to facilitate the diffusion of factors between adjacent cells. This functional network of gap junctions between oocyte and granulosa cells and between granulosa cells themselves maintain the follicle in a functionally integrated state and ultimately determines oocyte quality (figure 3). In addition to establishing physical contacts, the growing oocyte secretes members of the TGFβ family such as GDF9 and BMP15. These oocyte factors promote the formation of TZPs as well as granulosa cell proliferation and trigger the transition from primary to secondary stages of follicle development (figure 3B) (Gilchrist, et al., 2008). These oocyte secreted factors are important regulators of early follicle development and also modulate FSH-dependent granulosa cell cyto-differentiation, cumulus metabolism and expansion at later stages. The importance of oocyte secreted factors is highlighted in humans where mutations of *GDF9* and *BMP15* genes contribute to POI (Laissue, et al., 2006).

Whilst the relationship between the somatic cells and the oocyte is bidirectional and interdependent, recent studies have shown that the age of the somatic cell environment influences oocyte development and health. Aged mouse oocytes exposed to a young follicular environment regain functionality and show improved quality suggesting that the age of the somatic cells plays a critical role in oocyte quality (Wang, et al., 2024). The young follicular environment restored mitochondrial health in aged oocytes, reducing ROS levels and oxidative damage which are key factors contributing to oocyte aging. It is thought that younger granulosa cells are more efficient at forming an effective communication network with the oocyte and that is responsible for the rejuvenation effect, therefore targets to improve the communication network within the follicle need to be considered. It is possible that these may be a therapeutic target and that therapies directed at the somatic cells may have advantages compared to targeting oocytes directly.

As mentioned above, a significant factor in the reduction in oocyte quality with age is oxidative stress. The effects of ageing on the molecular mechanisms regulating the function of the human oocyte remain poorly understood, however, animal studies have shown that intraovarian levels of several members of the sirtuin family of regulators of mitochondrial function and oxidative stress decline with age. This decline is linked to declining levels of nicotinamide adenine dinucleotide (NAD^+^), an enzyme consisting of adenine and nicotinamide involved in the electron transport chain in mitochondria and an important signalling molecule which can affect cellular transcription of proteins and DNA repair. There is substantial interest in this potential approach to a wide range of age-related health conditions. Supplementation of NAD^+^ through the metabolic precursor nicotinamide mononucleotide (NMN) to aged mice resulted in improved egg quality with reduced abnormal meiotic chromosome spindles and increased numbers of pups born (Bertoldo, et al., 2020). Additionally, boosting NAD^+^ levels in mice treated with gonadotoxic chemotherapeutic drugs resulted in improved ovarian function, reflected in both oocyte number and number of pups (Ho, et al., 2024).

When the follicle reaches the multi laminar stage (figure 2) it forms a fluid filled space (antral cavity), leading to two functionally distinct populations of granulosa cells (mural and cumulus). The cumulus cells have a role in regulating oocyte maturation via paracrine regulation whilst the mural granulosa cells play a role in endocrine regulation and the synthesis of estrogens (Telfer, et al., 2023). Several layers of stromal-like cells around the follicular basal lamina differentiate to form theca layers and express LH receptors and steroidogenic enzymes in preparation for further follicle development and to respond to signals that result in ovulation. During the early stages of follicle development oocytes acquire the ability to resume meiosis with meiotic competence achieved at antral formation. Following this, oocytes need to be held in meiotic arrest until they receive maturation signals at ovulation. This is regulated via maintenance of elevated cAMP levels in the oocyte and cGMP from the granulosa cells and is dependent upon maintenance of good oocyte-somatic cell communication (figure 3). Resumption of meiosis is triggered by the surge of pituitary LH at ovulation. LH stimulates oocyte maturation via its action on the somatic cells and on the oocyte via expression of genes regulating EGF which influences nuclear maturation and oocyte competence (Telfer, et al., 2023).

The ever-increasing age at childbirth has brought oocyte chromosomal errors to the forefront of reproductive medicine practice. Even at peak fertile age (20-32 years old), around 20% of human oocytes are aneuploid, rising to >50% over the age of 32 (Gruhn, et al., 2019), resulting in a parallel increase in embryo aneuploidy. In understanding the mechanistic cause of this maternal age effect, a great deal of attention has focused on cohesin. As described above, cohesin entraps the two sister chromatids during DNA replication, which in mammalian oocytes takes place in the fetus. Cohesin is central to ensure chromosomes are properly distributed into the oocyte and experiments in model organisms have led to understanding of how cohesin supports chromosome segregation during optimal conditions (Duro and Marston, 2015). During meiotic prophase, cohesin supports the process of meiotic recombination where chromosomes are broken and resealed with the ultimate result of producing crossovers. Crossover mature into chiasmata, which are physical linkages between the homologs. Importantly, cohesin on chromosome arms stabilizes the chiasma and the cohesin-chiasma linkage allows homologous chromosomes to attach to spindle fibres from opposite poles by providing tension that resists microtubule forces. Ultimately, and ideally only when all chromosomes have properly attached in this manner, the enzyme separase becomes active and cuts cohesin, triggering the movement of homologs to opposite poles. However, cohesin is protected from separase-dependent cleavage in the pericentromere – the region around centromeres – by a protein known as shugoshin (SGO2). This pericentromeric cohesin is important for sister chromatids to attach to microtubules from opposite poles in meiosis II, and its subsequent loss at fertilisation by a second round of separase activation leads to even segregation of sister chromatids to opposite poles. Consequently, cohesin deterioration could lead to mis-segregation of chromosomes at meiosis I or II (figure 4). In practice, in humans, errors at both meiotic divisions are observed, but premature sister chromatid cohesion loss is predominant with advanced material age.

Substantial evidence from model organisms has implicated cohesin deterioration as a potential contributor to the increased aneuploidy in oocytes from women of advanced material age. Experiments in mouse oocytes found no evidence for cohesin turnover beyond meiotic prophase (Burkhardt, et al., 2016). The implication is that the cohesin laid down in the fetus must hold chromosomes together throughout the lifetime of the individual until ovulation of that oocyte, potentially 40+ years later in humans. However, recently cohesion establishment after meiotic S phase has been observed in the fruit fly, *Drosophila* (Haseeb, et al., 2024), which demonstrates that cohesin rejuvenation is possible. Nevertheless, many studies have observed that chromosomal cohesin reduces with age in mouse and human oocytes and that the distance between kinetochores of the sister chromatids increases with age, indicating a loss of cohesion (Charalambous, et al., 2023). Furthermore, partial depletion in many model systems leads to aneuploidy reminiscent of that observed with maternal age, supporting the idea that the deterioration of cohesin is a contributing factor. Furthermore, we recently found that the cohesin protector shugoshin declines at pericentromeres in human oocytes which led us to a model whereby weakened cohesin leads to reduced recruitment of shugoshin, which in turn leaves residual cohesin vulnerable to cleavage already in meiosis I (Mihalas, et al., 2024). This type of feedback loop could explain why the increase in oocyte aneuploidy with maternal age is not gradual but steepens after around age 35.

Key questions that remain to be addressed include why cohesin is particularly prone to deterioration? Is it because of a lack of turnover, or does turnover occur but inefficiently? And is there potential to activate cohesin rejuvenation pathways to restore functionality to aged oocytes and improve fertility, both natural and in assisted reproduction? While answers to these questions should be prioritised for further investigation, age-dependent effects on other components of the segregation machinery should not be neglected. Indeed, impaired checkpoint activity and defective kinetochores have also been implicated (Mihalas, et al., 2024).

## Figures and Tables

**Figure 1:**
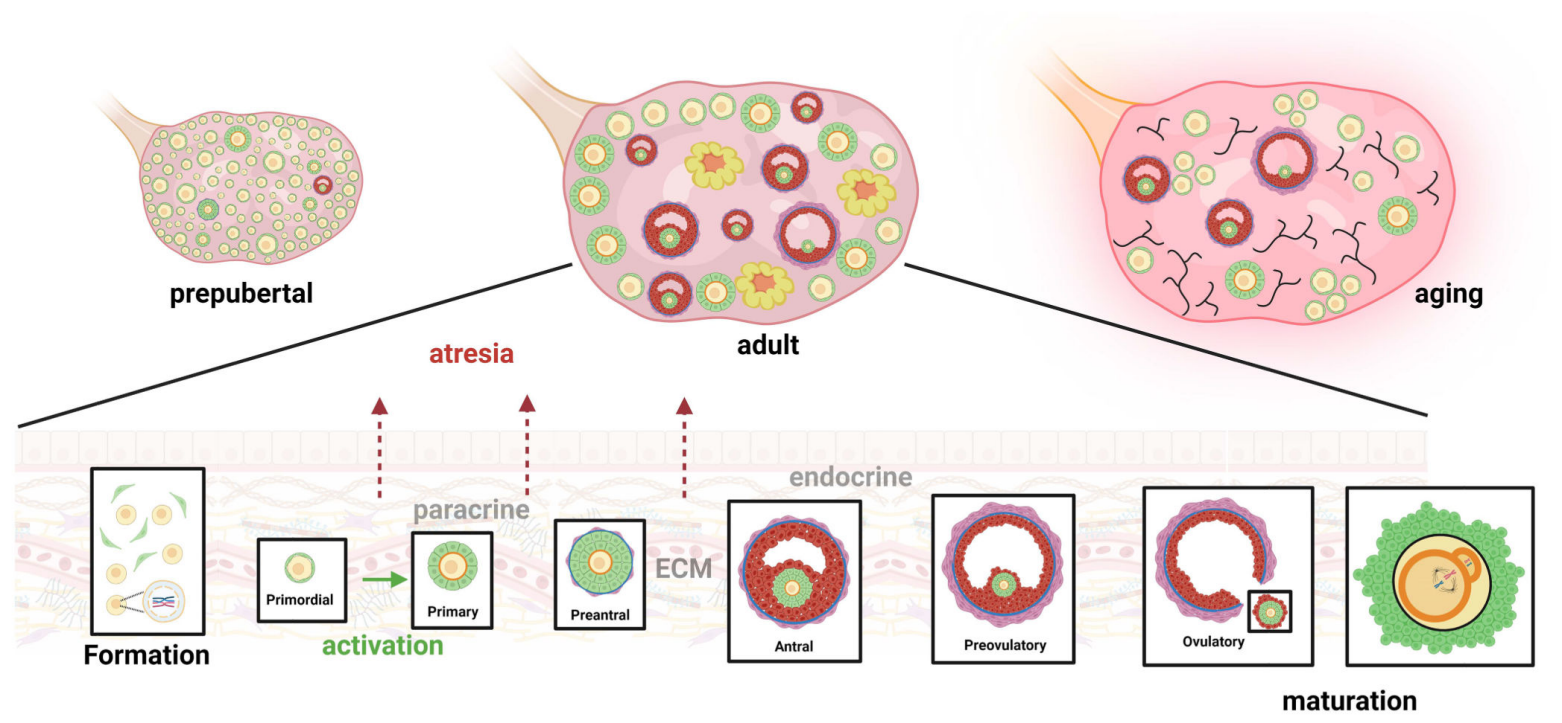
The dynamic microenvironment of the human ovary. The human ovary has the maximum endowment of follicles pre-pubertally and follicles initiate to grow and develop to early antral stages. Following the onset of puberty, all stages of follicle development up to ovulatory stages and corpora lutea are present all embedded within the ovarian stroma. During aging there is depletion of the primordial and growing follicle pool but ovulation continues until the menopausal transition. Aging leads to increased fibrosis of the ovarian stroma which affects the mechanical properties of the ovary that impact on follicle activation and growth.

**Figure 2:**
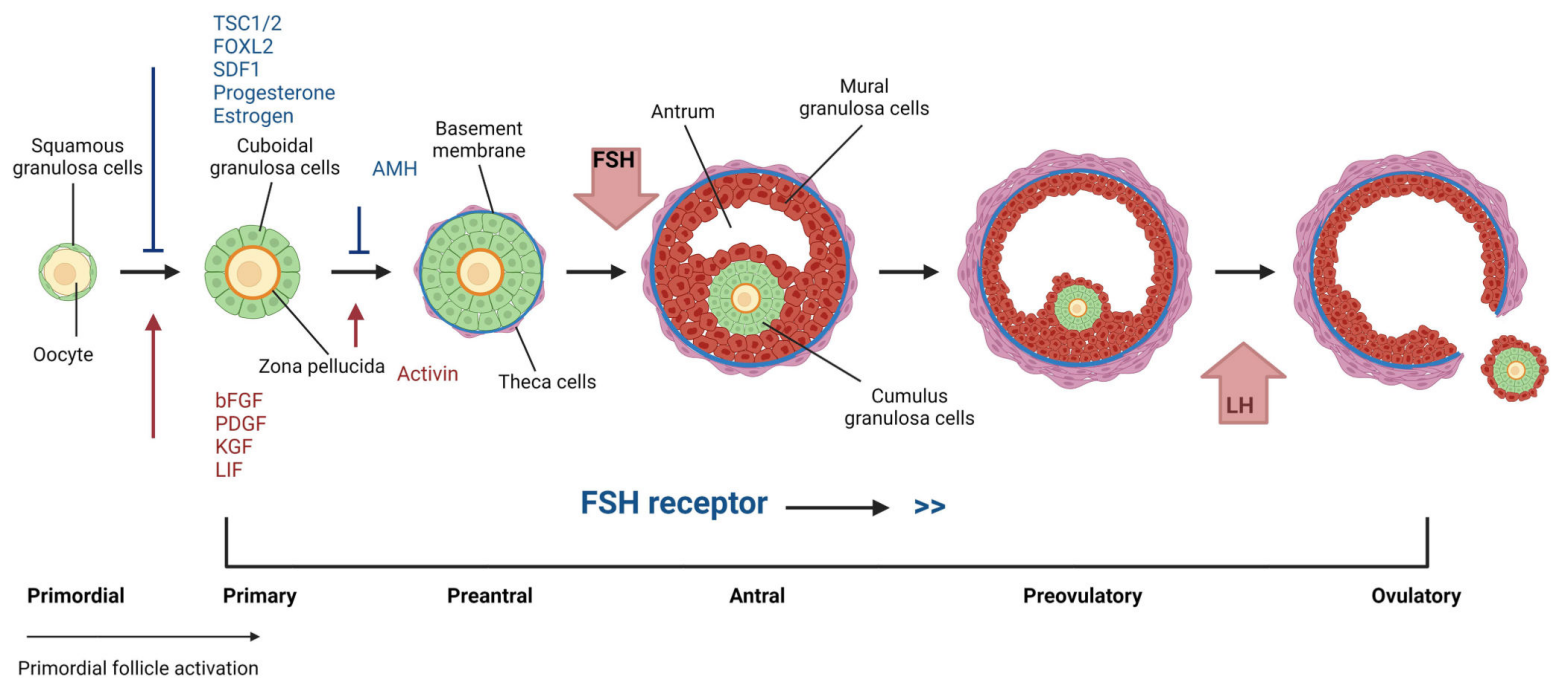
Development of ovarian follicles from primordial to pre-ovulatory. Primordial follicles consist of an oocyte arrested at the dictyate stage of Prophase 1 of meiosis surrounded by non proliferating flattened somatic (granulosa) cells. Several factors as listed have been implicated in the maintenance of quiescence (blue line and text) and activation of growth (red arrow and text). Activation of growth is characterized by the oocyte being surrounded by a complete layer of cuboidal granulosa cells (Primary stage). Paracrine factors such as activin stimulate proliferation of granulosa cells to form multi-laminar structures (pre-antral) which are surrounded by differentiated thecal cells. Pre-antral follicles undergo a morphogenetic transition to form a filled antral cavity with mural granulosa cells lining the wall of the follicle and cumulus granulosa cells surrounding the oocyte. Under the regulation of Follicle Stimulating Hormone (FSH) antral follicles undergo rapid growth to reach pre-ovulatory stages with the oocyte-cumulus complex being released at ovulation in response to Luteinising Hormone (LH) signalling.

**Figure 3:**
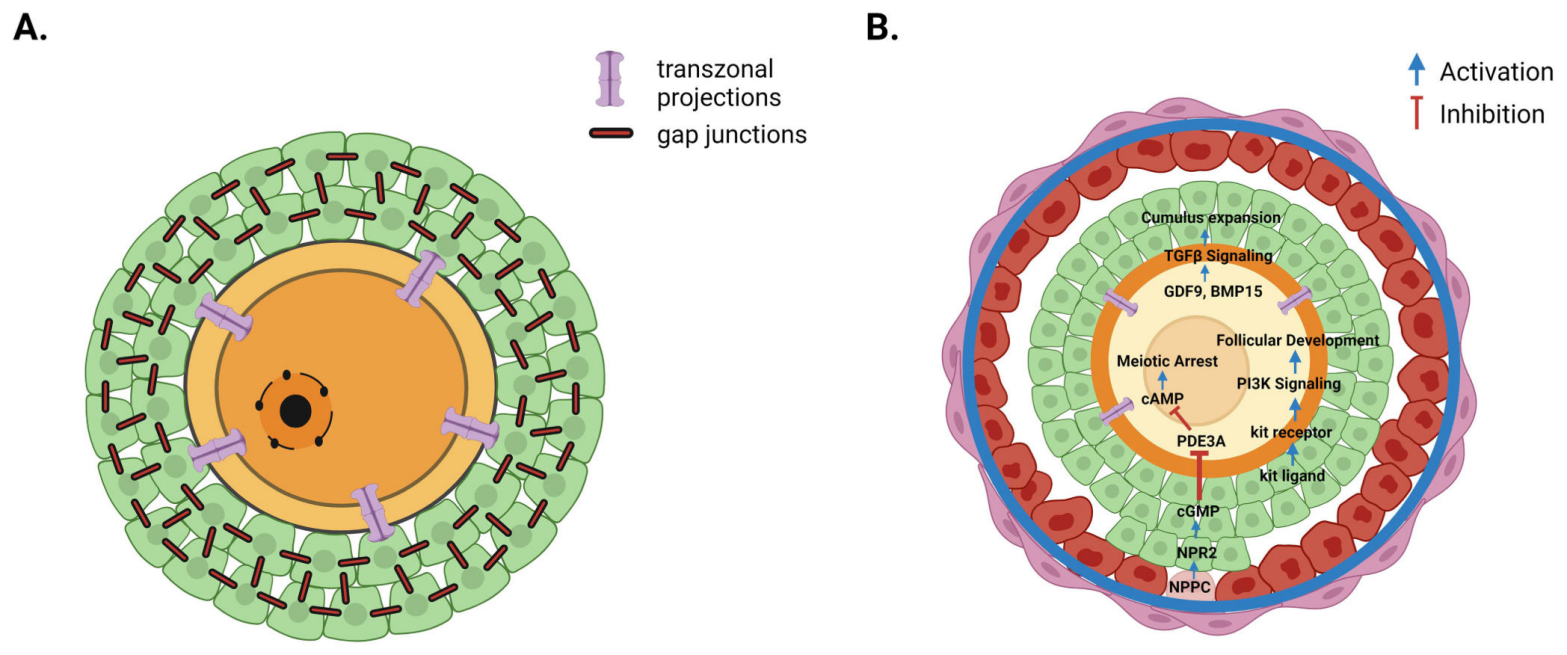
Communication network within the follicle. Communication between all cell types (oocyte, cumulus and mural granulosa cells and theca) within the growing follicle is facilitated through gap junctions and transzonal projections (TZPs) that form a syncytium to facilitate bi-directional communication (A) mediated by paracrine factors (B). Maintenance of this communication network is essential to support meiotic arrest, follicle and oocyte growth and granulosa cell differentiation through paracrine signalling (B). Re-initiation of meiosis requires the activation of maturation-promoting factor (MPF: a heterodimer composed of CDK1 and Cyclin B (B1, B2 and B3) and this needs to be inhibited to prevent premature resumption of meiosis. Inhibition is dependent upon intracellular cAMP levels being elevated in the oocyte with oocyte production of cAMP being the major pathway in regulating meiotic arrest with somatic cells playing an indirect role in maintaining elevated cAMP levels via cGMP from the granulosa cells inhibiting PDE3 activity within the oocyte (B). This is mediated via the natriuretic peptide C/natriuretic peptide receptor 2 (NPPC/NPR2) system. C-type natriuretic peptide (CNP) is produced in mural granulosa cells and its receptor NPR2 is expressed within cumulus granulosa cells. The production of cGMP in the granulosa cells inhibits the degradation of cAMP by inhibiting phosphodiesterase (PDE3) activity in the oocyte (B).

**Figure 4:**
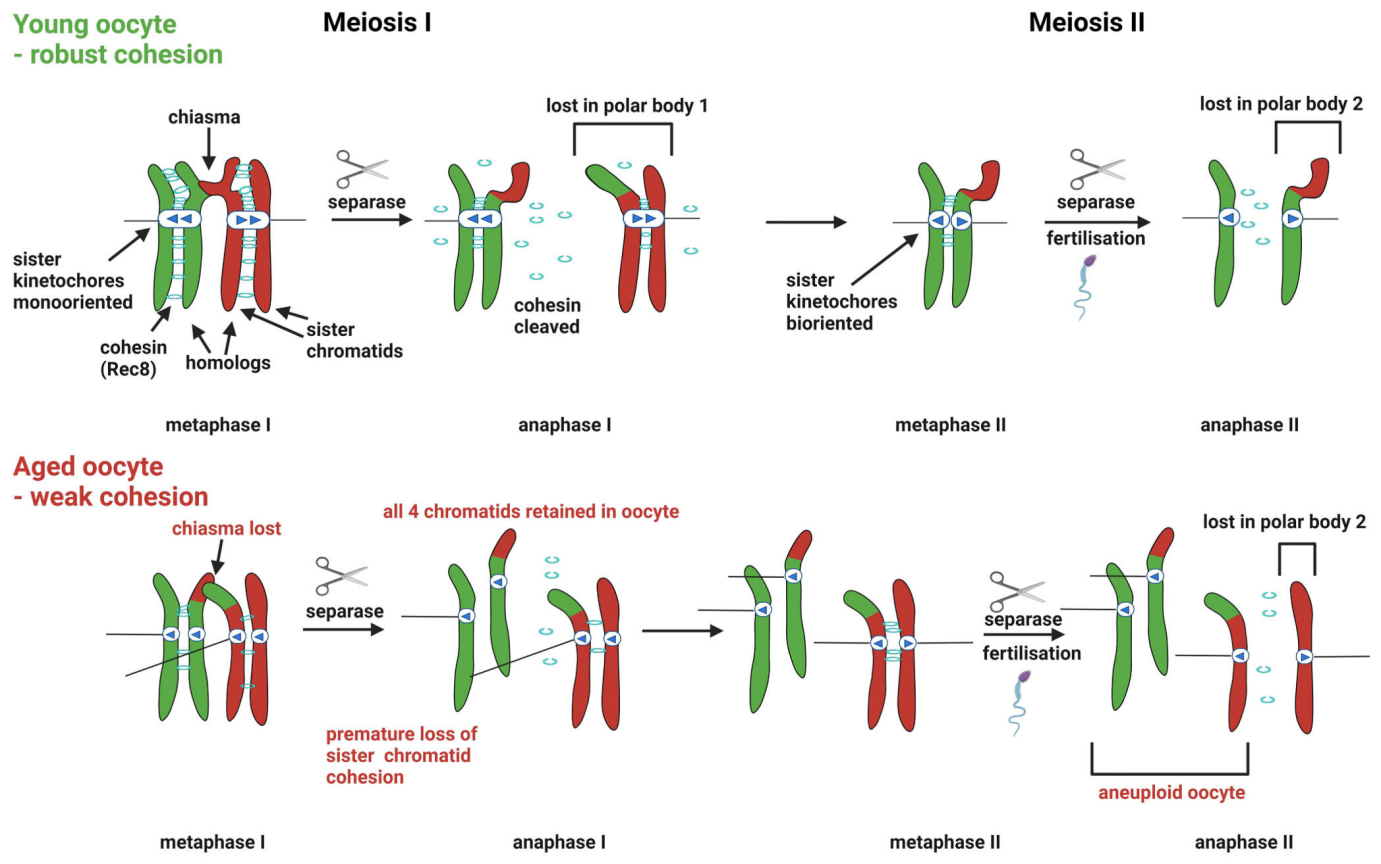
Error-free and erroneous meiotic chromosome segregation. Top schematic shows accurate segregation of a homologous chromosome pair in the two meiotic divisions. Bottom schematic shows the effect of cohesin deterioration in an aged oocyte.
